# The relationship between thyroid disorders and vitamin A.: A narrative minireview

**DOI:** 10.3389/fendo.2022.968215

**Published:** 2022-10-11

**Authors:** S. Capriello, I. Stramazzo, M. F. Bagaglini, N. Brusca, C. Virili, M. Centanni

**Affiliations:** ^1^ Department of Medico-Surgical Sciences and Biotechnologies, ‘‘Sapienza’’ University of Rome, Latina, Italy; ^2^ Endocrine Unit, Azienda Unità Sanitaria Locale Latina, Latina, Italy

**Keywords:** vitamin A, retinoic acid, thyroid autoimmunity, thyroid cancer, goiter, microbiota

## Abstract

The terms “vitamin A” and “retinoids” encompass a group of fat-soluble compounds essential for human nutrition. Some of them (retinol, retinal, 9-cis-retinoic acid, tretinoin, and 13-cis-retinoic acid) are fully natural, while others are synthetic compounds used mostly for therapeutic purposes. Some evidence indicates that the nutritional status of these retinoids (i.e., the presence or absence of deficiency) is able to modulate thyroid gland metabolism. Vitamin A deficiency is tightly correlated with structural and functional impairment of the thyroid gland and is often associated with iodine deficiency. Furthermore, retinoids are involved in different immune functions, as well as in the process of activation, proliferation, and differentiation of regulatory T cells (Treg). This is particularly significant given the high prevalence of thyroid autoimmune disorders, whose pathogenesis seems to be related to the altered homeostasis of regulatory T cells. Retinoids are also involved in the modulation of gene expression *via* their interaction with nuclear receptors, and they also act as cofactors in cell growth and differentiation. The ability of retinoic acid to increase iodine uptake and sodium-iodine symporter activity in human thyroid cancer cell lines suggests that some retinoids and their derivatives may be of use in the treatment of different thyroid tumors. This minireview summarizes the current knowledge on the link between nutritional intake of vitamin A and various thyroid disorders.

## Introduction

Micronutrient deficiency is a major public health problem, and marginal malnutrition is an issue of global significance. Macronutrient and micronutrient shortages often coexist and can interfere with normal human growth and development (1). In addition, micronutrient shortfall can lead to pathophysiological conditions that cause an increased need for and/or greater loss of nutrients (1). Among these nutrients, the role of vitamin A has been emphasized due to the key role it plays in fundamental life processes (2). The term “vitamin A” encompasses a group of fat-soluble compounds essential to human nutrition, which vary in their sources and bioavailability (2, 3). Similarly, the term “retinoids” includes both naturally occurring molecules and synthetic compounds that exhibit biological activities that are characteristic of vitamin A. Some of these compounds (including retinol, retinal, 9-cis-retinoic acid, ATRA [tretinoin], and 13-cis-retinoic acid) are fully natural. The second and the third generation of retinoids (including etretinate, acitretin, adapalene, tazarotene and bexarotene) are synthetic compounds derived from the natural ones and are mostly used for therapeutic purposes (2). Throughout this review, the terms “vitamin A” and “retinoid” will be used interchangeably.

Retinoic acid (RA), a multifunctional metabolite, is involved in most functions of vitamin A (2–4). Such involvement is primarily highlighted by the role of retinoic acid receptors (RARs and RXRs), which, besides homodimerization, can also heterodimerize (in the case of RXRs) with nuclear receptors such as the vitamin D receptor (VDR) and the thyroid hormone receptor (TR), forming a hormonal network (3, 4). Therefore, through these processes, retinoids can interact in multiple pathophysiological functions and have pleiotropic effects within the human body (3, 4). The retinoids’ mechanisms of action are strongly time- and dose-dependent. Retinoids are involved in epithelial cell proliferation and differentiation, in the immune response, and in the processes of morphogenesis and carcinogenesis. The retinoid system acts as a true endocrine system, in which retinoic acid is the receptor ligand leading to hormonal activity (2–4). The overall distribution of dietary forms of vitamin A and their transformation into signaling retinoic acid molecules thus represents a hormonal network (2, 3).

Among the micronutrients, vitamin A seems to play an especially significant role due to its ability to modulate thyroid homeostasis alone or in interaction with other micronutrients, particularly with iodine (2, 5, 6). These compounds share a retinoid chemical structure and can be supplied in the diet *via* both animal (all-trans retinol or retinyl esters) and vegetable (carotenoids) sources (2, 3).

The complex process of absorption, distribution, and metabolism of vitamin A, and its natural or synthetic derivatives, shares characteristics with the corresponding process for thyroid hormones (7). Moreover, the absorption of retinoids follows the pathway of lipids: due to their hydrophobicity, these compounds require a binding protein so that they can be transported within the bloodstream (2, 7). Retinol and the retinol binding protein (RBP) create a tertiary complex with transthyretin (TTR), which is also involved in the transport of thyroid hormones in the blood (4–7). In a study in TTR knockout mice, plasma levels of both thyroid hormones and retinol were significantly reduced (7–9), though their tissue levels were normal (9). Furthermore, the cellular effects of vitamin A compounds and thyroid hormones appear to be associated with gene regulation and modulation due to their interaction with nuclear receptors (2, 4).

This minireview summarizes the current knowledge on the link between nutritional intake of vitamin A and various thyroid disorders.

## Vitamin A and thyroid pathophysiology

Retinoid nutritional status is able to modulate thyroid gland homeostasis. Vitamin A deficiency (VAD) appears to be closely related to thyroid gland impairment, due also to its frequent association with iodine deficiency (ID) (3, 4, 6, 10). Indeed, retinoids seem to play a role in the development and maturation of the thyroid cell phenotype (4).

Several animal studies using mouse models have shown that VAD is related to reduced iodine uptake, impaired coupling of iodothyronines, and reduced thyroglobulin synthesis, which can lead to thyroid hypertrophy and goiter and to a decreased intrathyroidal pool of T3 and T4 (10–15). The hypothesis that vitamin A deficiency, in the presence of protein-calorie malnutrition, might impair the glycosylation of thyroglobulin (Tg) was proposed by Ingenbleek Y in 1983 (12). Through the analysis of the intracellular Tg glycosylation and iodination processes, Ingenbleek postulated that vitamin A depletion would impair the endogenous retinyl-phosphate-mannose synthesis and, therefore, the normal thyroglobulin mannosylation in rats. As a consequence, the Tg would feature a steric hindrance, with abnormal disulphide bonds closure, reduced coupling reactions, and decreased production of thyroid hormones (12). Since then, the discovery of the nuclear retinoid receptors, and of the modulatory intracellular binding proteins carrying retinoids to these receptors, has shed light on these compounds and extended the knowledge of their biological activity. Ingenbleek’s hypothesis has therefore been abandoned.

Some effects of vitamin A seem to be associated with a modulation exerted by retinol in the TSH-secreting pituitary function (13–15). The TSHß gene transcription in the pituitary gland may be suppressed by both thyroid hormone receptors and RXR (10, 15). A study in VAD mice showed that TSH levels had increased twice as much despite increasing total thyroxine levels, suggesting a role for VAD in creating a sort of resistance to thyroid hormones, reducing the risk of hypothyroidism (14). Moreover, the effect of VAD on the thyroid axis is exacerbated when associated with ID, in both animals and humans (3, 4, 10, 14). In concomitant VAD and ID animal models, it was demonstrated that vitamin A supplementation (VAS), with or without iodine supplementation, was able to reduce thyroid volume and TSH serum levels and increase thyroid iodine uptake (10, 14–16).

Vitamin A status may even affect thyroid homeostasis by interfering with the peripheral metabolism of thyroid hormones (2, 10). In fact, in mice models, it was observed that VAD reduces T3 binding and uptake by tissues and decreases hepatic conversion of T4 to T3, thus increasing the blood levels of both total and free T4 and T3 (10, 16–18). Furthermore, the effect of VAD on thyroid homeostasis may be mediated at least partly through shared transport proteins (3, 8, 9), i.e., TTR, which carries about 10%–15% of thyroid hormones (19). As previously described, RBP forms a complex with TTR, and this link prevents renal clearance of vitamin A and enhances its delivery to tissues (12, 20). Animal models have suggested that the binding capacity and affinity of TTR to thyroid hormones may be modified by interaction with RBP (10, 20). Moreover, in VAD animal models, it has been shown that even the hepatic release of RBP was decreased despite an unchanged serum level of TTR (10, 20).

The role of VAD and VAS in human thyroid functions has been studied by several authors (2–4, 10). Most of these studies were carried out in developing countries, where multiple or selected nutrient and micronutrient deficiencies occur. More specifically, in some areas VAD is concurrent with ID. A recent randomized study conducted on Iranian levothyroxine-replaced patients showed that, upon dietary supplementation with zinc, magnesium, and vitamin A for 3 months, the levels of free thyroxine (FT4) increased significantly, while those of C-reactive protein decreased (21). However, due to the concomitant supplementation, the specific role of each micronutrient cannot be distinguished.

In the past, some observational studies conducted in African populations (Senegalese adults, Ethiopian children) showed that there was a direct negative correlation between VAD and thyroid volume (22–24), without any variation in thyroid function. Furthermore, an Italian study carried out on a healthy population (20–107 years of age) showed no significant correlations between serum retinol levels and thyroid function, particularly in the younger age groups and in the elderly (25), even though higher retinol levels were associated with a decrease in TSH and FT4 in the oldest-old subjects (25). However, this category of patients may present several different biochemical profiles, independent of retinol status (26, 27).

The effect of VAS in humans with concomitant VAD and ID was investigated in two randomized studies conducted by Zimmerman M.B. et al. on Moroccan and South African children (28, 29). The Moroccan study found that in children severely affected by iodine deficiency disorders, severe VAD is a predictor of increased thyroid volume. The authors concluded that in children with both iodine and vitamin A deficiency, concurrent VA supplementation improves iodine efficacy (28). These findings indicate that supplementation of VA in severe ID populations may activate a compensatory effect of TSH and TT4 production, thus decreasing thyroid volume and the risk of hypothyroidism (10, 28). In the study carried out in South Africa (29), it was shown that iodine prophylaxis is effective in controlling ID independently of VAD, and that VA supplementation has an additional benefit for the thyroid, in addition to the treatment of VAD. In fact, VAS suppresses TSH-β gene expression, limiting TSH stimulation of the gland and thereby reducing the risk of goiter. The authors suggest that, as in the animal models (11, 14–17), vitamin A nutritional status may modify thyroid sensitivity to TSH and/or the peripheral metabolism of thyroid hormones (10, 24, 29). This is in keeping with the results obtained by Elnour et al. (30), who observed a TT4 increase in VAD in Sudanese children, despite iodine sufficiency.

Again, different findings were reported in an Italian study conducted by Ceresini et al. (31) on 11 healthy subjects. The authors described a substantial lack of effectiveness of 10-day VA supplementation for modifying TSH levels. However, the short observation period and the small sample size prevent drawing definitive conclusions. A relatively recent Iranian study by Farhangi et al. observed that, in healthy premenopausal obese and non-obese women, 4 months of VAS was able to reduce TSH and increase T3 levels (possibly due to a hepatic effect on the conversion of thyroid hormones), reducing the risk of subclinical thyroid dysfunction (32). Despite some differences, when taken together, these findings suggest that fluctuations in vitamin A levels may affect thyroid homeostasis and, consequently, its morphology and function, *per se* and independently from ID.

## Vitamin A, Hashimoto’s thyroiditis, and gut microbiota

Retinoids are involved in various immune functions and in the process of activation, proliferation, and differentiation of regulatory T cells (Treg) due to the presence of retinoid receptors on lymphocytes (33–35). Vitamin A seems to be able to modulate the differentiation of Treg in the presence of high levels of interleukin (IL)-2 and to modify the release of transforming growth factor beta (TGFß) by these immune cells (33, 35). Retinoic acid receptor α (RARα) seems to be crucial for the modulation of T-cell responses, as deletion of RARα in CD4^+^ T cells had effects similar to those observed with VAD-induced disruption of T-cell homeostasis (e.g., Treg-Th1 [T helper cell 1]/Th17 imbalance) (36). By decreasing interferon-γ and IL-17, retinoic acid suppresses the inflammatory Th1/Th17 responses. It also enhances IL-4, IL-10, IL-22, and TGFß, and by modulating mucin gene expression and the production of intestinal immunoglobulin A, it ensures intestinal integrity (35). Through these mechanisms, RA may counteract different pathologies, including type 1 diabetes, inflammatory bowel disease, and multiple sclerosis (36).

Hashimoto’s thyroiditis (HT) is the most frequently diagnosed autoimmune disease worldwide. It is also an exemplar of an organ-specific auto-aggressive disorder (37). In this disease, an early Th17-driven inflammatory phase is followed by a typical Th1 polarization (37). It may also occur in the context of polyautoimmunity, in which case Th2 polarization has also been described (38).

A recent study examined the association between vitamin A–related genes and the susceptibility to, and prognosis of, HT (39). Notably, the authors observed that some of the polymorphisms in the CYP26B1 gene were associated with the severity of HT, while those in the RAR gene seemed to be associated with Th17 polarization and with HT susceptibility (39).

In patients with HT, dysbiosis plays an important role in the autoimmune process and affects the proper absorption of micronutrients in numerous ways (40). The variation in microbiota composition, as well as the leakiness of the gut barrier, represents a major trigger of autoimmunity (41). Although further specific studies are warranted, the overall role of the microbiota in determining autoimmune processes, along with the evidence that RA demonstrates protective effects on the intestinal barrier, makes a regulatory role for vitamin A in Hashimoto’s thyroiditis more than a simple hypothesis (39–41).

## Vitamin A and thyroid cancer

As mentioned above, retinoids are involved in the modulation of gene expression by their interaction with nuclear receptors, and they act as cofactors in cell growth and differentiation (42, 43). The mechanisms involved act primarily at the transcriptional level (by enhancing transcription of target genes and of genes encoding transcription factors and signaling proteins) or at the epigenetic level (by modifying the interactions between DNA and proteins) (42). Some retinoids and their derivatives have been used and/or proposed in the treatment of different neoplasms (43), with conflicting results (43–45).

*In vitro* and *in vivo* studies have evaluated the effect of the nutritional status of vitamin A in patients with thyroid cancer. The scientific rationale regarding the role of vitamin A compounds in thyroid cancer was based on the ability of retinoic acid to increase iodine uptake and sodium-iodine symporter activity in human thyroid cancer cell lines (46, 47). Based on this evidence, several studies have tried to analyze the effects of vitamin A on advanced thyroid cancer or radioactive iodine–refractory (RAI-R) differentiated thyroid cancer (48–51). A recent study found that elevated serum levels and/or oral intake of vitamin A and vitamin E were associated with a less aggressive clinical presentation and better prognosis in patients with papillary thyroid carcinoma (52). These authors also suggested that adequate vitamin A intake might be able to prevent extra-thyroidal extension and lymph node metastasis (52). Notably, one small group of patients with radioiodine-refractory thyroid cancer was treated with 13-cis-retinoic acid followed by iodine-131 treatment; after this “re-differentiation therapy,” 2 out of 3 patients whose cancers featured the BRAF V600E mutation showed a response expressed in terms of tumor size, serum Tg levels, and iodine uptake (53). Unfortunately, despite the interesting scientific basis, no studies have shown a definitive usefulness of vitamin A in thyroid cancer. Furthermore, one of the most recent reviews on this topic showed that only a minority of patients with RAI-R differentiated thyroid cancer respond well to retinoid treatment (54).

The effect of retinol on thyroid C cells is still unknown (4). An *in vitro* study on the rat C cell line CA-77 showed a decrease in calcitonin release due to retinoid acid 9-cis (55), while all-trans retinoic acid did not seem to have an effect on a human medullary thyroid carcinoma cell line (56). Furthermore, no data suggest the physiological presence of retinoids in thyroid parafollicular cells (3).

The main limitations of this minireview are the limited quantity of evidence and the fact that some of the more interesting findings are not recent.

## Concluding remarks

**Figure 1:**
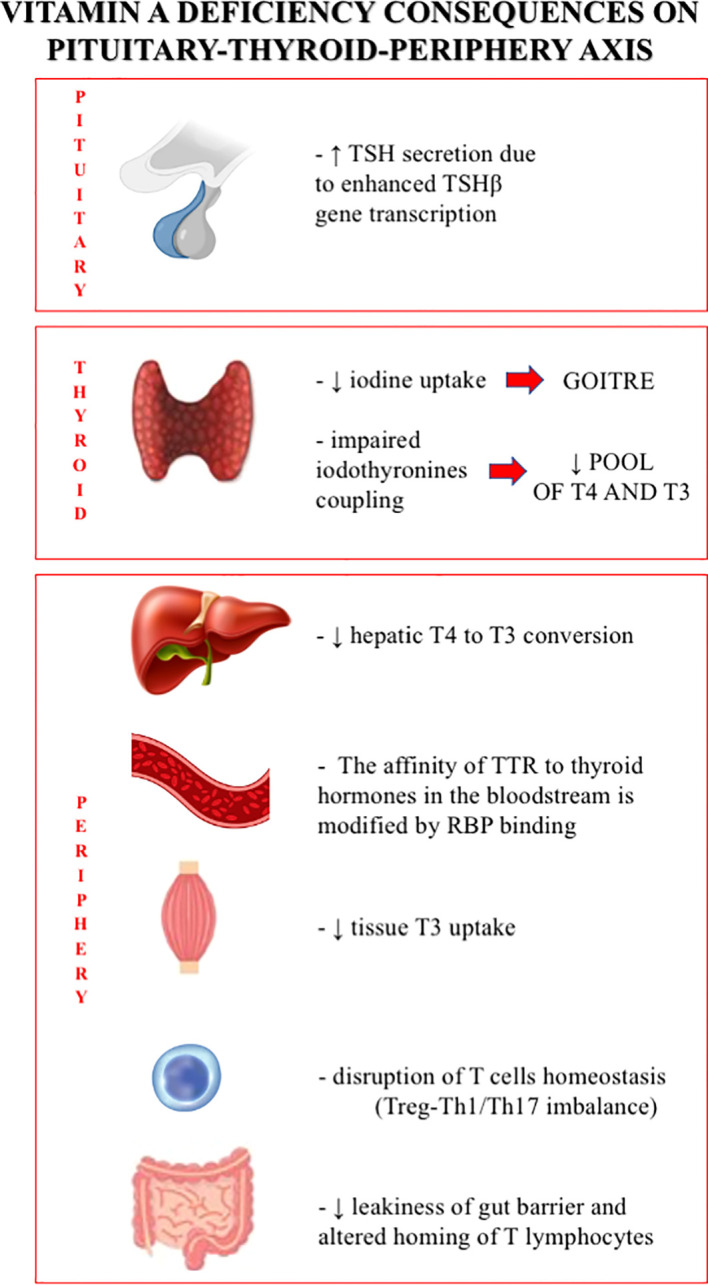
Vitamin A deficiency consequences on pituitary-thyroid-periphery axis.

Despite the above-mentioned limitations, vitamin A and its active derivatives appear to have a meaningful effect on thyroid morphology, function, and homeostasis (see **Figure 1**). The roles of vitamin A in the development of goiter, in the treatment of thyroid cancer, and in the modulation of the adaptive immune response have been the main focus of our discussion. The need for further studies on this topic is warranted.

## Author contributions

CM and VC designed the study and contributed to the manuscript; CS and SI wrote the manuscript. BN and BM revised the pertinent literature and revised the manuscript. All authors revised and approved the final manuscript.

## Conflict of interest

The authors declare that the research was conducted in the absence of any commercial or financial relationships that could be construed as a potential conflict of interest.

## Publisher’s note

All claims expressed in this article are solely those of the authors and do not necessarily represent those of their affiliated organizations, or those of the publisher, the editors and the reviewers. Any product that may be evaluated in this article, or claim that may be made by its manufacturer, is not guaranteed or endorsed by the publisher.
